# Increased di-(2-ethylhexyl) phthalate exposure poses a differential risk for adult asthma clusters

**DOI:** 10.1186/s12931-024-02764-8

**Published:** 2024-03-23

**Authors:** Yuan-Ting Hsu, Chao-Chien Wu, Chin-Chou Wang, Chau-Chyun Sheu, Yi-Hsin Yang, Ming-Yen Cheng, Ruay-Sheng Lai, Sum-Yee Leung, Chi-Cheng Lin, Yu-Feng Wei, Yung-Fa Lai, Meng-Hsuan Cheng, Huang-Chi Chen, Chih-Jen Yang, Chien-Jen Wang, Huei-Ju Liu, Hua-Ling Chen, Chih-Hsing Hung, Chon-Lin Lee, Ming-Shyan Huang, Shau-Ku Huang

**Affiliations:** 1 National Institute of Environmental Health Sciences, National Health Research Institutes, No.35, Keyan Road, Zhunan, Miaoli County, 35053 Taiwan; 2 National Center for Geriatrics and Welfare Research, National Health Research Institutes, Miaoli, Taiwan; 3 Division of Pulmonary and Critical Care Medicine, Department of Internal Medicine, Kaohsiung Chang Gung Memorial Hospital, Chang Gung University College of Medicine, Kaohsiung, Taiwan; 4 Department of Public Health, Kaohsiung Medical University, Kaohsiung, Taiwan; 5 Division of Pulmonary and Critical Care Medicine, Department of Internal Medicine, Kaohsiung Medical University Hospital, Kaohsiung Medical University, Kaohsiung, Taiwan; 6 Department of Internal Medicine, School of Medicine, College of Medicine, Kaohsiung Medical University, Kaohsiung, Taiwan; 7 National Institute of Cancer Research, National Health Research Institutes, Tainan, Taiwan; 8 Department of Mathematics, Hong Kong Baptist University, Hong Kong, China; 9 Division of Chest Medicine, Kaohsiung Veterans General Hospital, Kaohsiung, Taiwan; 10 Chest Division, Department of Internal Medicine, Antai Medical Care Cooperation Antai Tian-Sheng Memorial Hospital, Ping-Tung, Taiwan; 11 Department of Internal Medicine, E-Da Cancer Hospital, I-Shou University, Kaohsiung, Taiwan; 12 Division of Chest Medicine, Department of Internal Medicine, E-Da Hospital, I-Shou University, Kaohsiung, Taiwan; 13 Department of Respiratory Therapy, College of Medicine, Kaohsiung Medical University, Kaohsiung, Taiwan; 14 Division of Pulmonary and Critical Care Medicine, Department of Internal Medicine, Kaohsiung Municipal Siaogang Hospital, Kaohsiung Medical University, Kaohsiung, Taiwan; 15 Department of Pediatrics, Kaohsiung Medical University Hospital and College of Medicine, Kaohsiung Medical University, Kaohsiung, Taiwan; 16 Department of Pediatrics, Kaohsiung Municipal Siaogang Hospital, Kaohsiung Medical University, Kaohsiung, Taiwan; 17 Department of Marine Environment and Engineering, National Sun Yat-Sen University, Kaohsiung, Taiwan; 18 Johns Hopkins University School of Medicine, Baltimore, MD USA; 19 Department of Pediatrics, Kaohsiung Municipal Hsiao-Kang Hospital, Kaohsiung Medical University, Kaohsiung, Taiwan

**Keywords:** DEHP, HEL, Comorbidities, Asthma phenotype, Inflammatory markers

## Abstract

**Background:**

DEHP, a common plasticizer known for its hormone-disrupting properties, has been associated with asthma. However, a significant proportion of adult asthma cases are “non-atopic”, lacking a clear etiology.

**Methods:**

In a case-control study conducted between 2011 and 2015, 365 individuals with current asthma and 235 healthy controls from Kaohsiung City were enrolled. The control group comprised individuals without asthma, Type 2 Diabetes Mellitus (T2DM), hypertension, or other respiratory/allergic conditions. The study leveraged asthma clusters (Clusters A to F) established in a prior investigation. Analysis involved the examination of urinary DEHP metabolites (MEHP and MEHHP), along with the assessment of oxidative stress, sphingolipid metabolites, and inflammatory biomarkers. Statistical analyses encompassed Spearman’s rank correlation coefficients, multiple logistic regression, and multinomial logistic regression.

**Results:**

Asthma clusters (E, D, C, F, A) exhibited significantly higher ORs of MEHHP exposures compared to the control group. When considering asthma-related comorbidities (T2DM, hypertension, or both), patients without comorbidities demonstrated significantly higher ORs of the sum of primary and secondary metabolites (MEHP + MEHHP) and MEHHP compared to those with asthma comorbidities. A consistent positive correlation between urinary HEL and DEHP metabolites was observed, but a consistent negative correlation between DEHP metabolites and selected cytokines was identified.

**Conclusion:**

The current study reveals a heightened risk of MEHHP and MEHP + MEHHP exposure in specific asthma subgroups, emphasizing its complex relationship with asthma. The observed negative correlation with cytokines suggests a new avenue for research, warranting robust evidence from epidemiological and animal studies.

**Supplementary Information:**

The online version contains supplementary material available at 10.1186/s12931-024-02764-8.

## Background

Phthalates, recognized as environmental contaminants and endocrine-disrupting chemicals, have demonstrated adverse effects on reproductive and developmental systems [1–4]. Recent studies indicate that metabolites of di-(2-ethylhexyl) phthalate (DEHP), a common plasticizer, may play a role in immune regulation and are associated with respiratory and allergic diseases [5, 6]. Furthermore, DEHP exposure has been linked to an increased prevalence of childhood allergic asthma and adult asthma [7–9]. However, the connection between DEHP exposure and adult asthma lacks clarity, especially considering that a substantial portion of adult asthma cases is classified as “non-atopic” with an unclear etiology.

In asthma, genetic factors alone cannot account for the heightened morbidity, emphasizing the crucial role of environmental factors in triggering or exacerbating the disease. Given the heterogeneity of asthma as a chronic condition, exploring variations in DEHP exposure across different phenotypes could enhance our understanding of its impact on asthma heterogeneity. Building upon our prior research, we have illustrated diverse exposure risks to ambient air pollutants among current asthma patients with six distinct phenotypes. These phenotypes are defined by 18 demographic and clinical variables, resulting in Cluster A (older non-atopic and non-smoker females with late-onset asthma), Cluster B (primarily older atopic and non-smoker females), Cluster C (older ex-smoking males with second-hand smoke exposure), Cluster D (older non-smoking atopic females with high BMI and poor lung function), Cluster E (young males and current smokers with early onset and low Asthma Control Test (ACT) scores), and Cluster F (young, atopic, non-smoker males with early onset), laying a crucial foundation for unraveling the correlation between the environment and asthma [10, 11].

Rodent studies support the notion that phthalates, acting as adjuvants at environmentally relevant doses, can induce respiratory and inflammatory effects in the presence of an allergen [12]. Cellular studies have shown that phthalates may modify both innate and adaptive immune responses [12]. Additionally, adult asthma is often associated with comorbidities, and asthma and type 2 diabetes mellitus (T2DM) are two common chronic diseases with an increasing incidence, possibly attributed to low-grade systemic inflammation and the use of corticosteroids and other medications [13]. However, research on the comorbidity of asthma with other diseases is currently limited. Ongoing efforts by researchers seek to deepen our understanding of the interrelationships between asthma and various illnesses, aiming to facilitate the development of more comprehensive and effective treatment methods and management strategies.

DEHP is widely employed to enhance the plasticity of plastic products, with approximately 97% of DEHP utilized in PVC products. Throughout the manufacturing, usage, and disposal phases, DEHP may be released into the environment due to processes like heating or wear and tear. Exposure to DEHP can arise from various sources, including dietary habits, daily life products, medical procedures, and drug use. In the general population, the primary routes of DEHP exposure are ingestion and inhalation. Significantly, the sources of DEHP exposure vary among different age groups, leading to corresponding differences in exposure concentrations [14].

In Taiwan, phthalates, including DEHP, have been identified in the human body [15, 16], food [17], and soil [18]. Nevertheless, a notable gap persists in establishing the threshold for DEHP exposure in the context of asthma disease. This gap is primarily attributed to unclear sources of exposure, individual variations, and the heterogeneity of asthma.

Continuing from our prior research [10, 11], this study employed the phenotypic clusters established in the previous investigation to examine variations in DEHP exposure among different phenotypes of asthma patients and further explored DEHP exposure levels across asthma and its comorbidities. This assessment utilized primary and secondary DEHP metabolites, MEHP and MEHHP, respectively, as markers of exposure, along with a panel of physiological biomarkers to investigate associations. The goal is to enhance our understanding of the health effects of DEHP exposure on asthma heterogeneity and its comorbidities.

## Methods

### Study participants

The case-control study comprised 365 individuals with “current asthma” attending clinical visits and 235 healthy controls undergoing routine checkups from Kaohsiung City in Taiwan, recruited between 2011 and 2015 (Figure. A.1). Healthy controls were individuals aged between 20 and 65 years without asthma, Type 2 Diabetes Mellitus (T2DM), hypertension, and other respiratory and allergic diseases. The cases were selected through simple random sampling from a population of 1,163 asthmatic subjects, who were phenotypically categorized into six clusters (for details, see Wu et al., 2022). To continue from our previous research [10, 11], this study employed the phenotypic clusters established in previous study to explore the variations in DEHP exposure among different phenotypes of asthma patients. Cluster A was a group of older non-atopic and non-smoker females with late onset asthma, while Cluster B included primarily older atopic and non-smoker females. Cluster C included older ex-smoking males with second-hand smoke exposure, and Cluster D consisted of older non-smoking atopic females with high BMI and poor lung function. Cluster E included young male and current smokers with early onset and low Asthma Control Test (ACT) scores, while Cluster F consisted of young, atopic, non-smoker males with early onset. The study was approved by the ethics committees of the National Health Research Institutes in Miaoli, Taiwan, and the respective recruiting hospitals. Moreover, consent to participate was obtained from all participants involved in the study.

### Data collection

To ensure the consistency and reliability of sample collection, processing, and storage, specific measures were implemented. Standardized procedures were utilized to collect urine and blood samples during a single visit by participants, minimizing potential variability. Venous blood was drawn using vacuum blood collection tubes (BD Vacutainer EDTA Blood Collection Tubes with K2 EDTA, Becton, Dickinson and Company, Franklin Lakes, NJ, US), while urine was collected in sterilized centrifuge tubes (BD Falcon Conical Tubes, Becton, Dickinson and Company, Franklin Lakes, NJ, US). These stringent procedures guaranteed standardized and controlled conditions for subsequent analysis. Following collection, all samples were promptly processed, separated into 15 c.c. sterilized centrifuge tubes for both plasma and urine, and then stored at -80 °C.

### DEHP metabolites

The urinary metabolites of di(2-ethylhexyl) phthalate (DEHP), namely Mono(2-ethylhexyl) phthalate (MEHP) and mono(2-ethyl-5-hydroxyhexyl) phthalate (MEHHP), underwent analysis via liquid chromatography-tandem mass spectrometry (LC-MS/MS). The LC-MS/MS method utilized for examining DEHP metabolites has been previously published [19, 20], with the limit of detection (LOD) for MEHP and MEHHP being 0.7 and 0.3 ng/mL, respectively.

### Oxidative stress, sphingolipid metabolites, and inflammatory biomarkers

Urinary concentrations of Nε-(hexanoyl)-lysine (HEL) and 4-hydroxynonenal (4-HNE), an early and a late lipid peroxidation products [21, 22], respectively, were measured using the HEL ELISA kit (JaICA, Nikken SEIL Co., Shizuoka, Japan) and the OxiSelect HNE Adduct Competitive ELISA Kit (Cell Biolabs, Inc., CA, USA). The minimum levels assessed for HEL and 4-HNE were 9.63 nmol/L and 0.005 µg/mL, respectively. Plasma concentrations of sphingosine-1-phosphate (S1P) and ceramide-1-phosphate (C1P) were analyzed using ELISA kits (MyBiosource, MBS069092 and MBS2601367, respectively; CA, USA) with detection limits of 1 ng/mL and 0.5 ng/mL for S1P and C1P, respectively. In addition, plasma concentrations of inflammatory biomarkers including IL-1β, IL-6, IL-8, IL-10, IL-13, IL-17 A, IFN-γ, MCP-1, and MIP-1β were measured using the Bio-Plex Pro Human Cytokine Assay and Bio-Plex 200 Systems (Bio-Rad) by the service platform of SUU-FLOWER Co., Taiwan. The minimum levels assessed for IL-1β, IL-6, IL-8, IL-10, IL-13, IL-17 A, IFN-γ, MCP-1, and MIP-1β were 0.03, 0.23, 1.33, 0.20, 0.04, 0.06, 0.13, 0.21, and 3.48 ng/mL, respectively. The oxidative stress, sphingolipid metabolites, and inflammatory biomarkers were evaluated in triplicate according to the manufacturers’ provided experimental protocols. These analyses were conducted following the official instructions, demonstrating a coefficient of variation below 10%.

### Statistical analysis

Asthma phenotypic clusters were classified using t-SNE (t-distributed Stochastic Neighbor Embedding) and implemented in R version 3.4.2 with the Rtsne package (https://cran.rproject.org/web/packages/tsne/index.html), which included 18 clinical and demographic parameters as previously described [23]. t-SNE is a powerful technique for visualizing and analyzing intricate high-dimensional data. Its strength lies in preserving local data structure, making it ideal for uncovering underlying patterns and clusters within such data. This method transforms complex data into a more manageable, low-dimensional form, facilitating the identification and understanding of data patterns and similarities. Hence, t-SNE is highly valuable for revealing hidden structures and clustering in data. To compare medians and proportions between cases and controls, or among phenotypic clusters and controls, we employed the Mann-Whitney U, Kruskal-Wallis H, and Chi-square tests. These tests assessed differences in continuous variable distributions between two or more groups (Mann-Whitney U and Kruskal-Wallis H) and differences in categorical variable distributions among multiple groups (Chi-square test). After performing the Kruskal–Wallis H test, we adopted to utilize Bonferroni test for post-hoc analysis. Spearman’s rank correlation coefficients were used to evaluate the correlation between the concentrations of urinary DEHP metabolites and oxidative stress, sphingolipid metabolites, and inflammatory biomarkers. After adjusting for potential confounders such as age, BMI, atopy, education, and household income, multiple logistic regression was used to assess the association between urinary DEHP metabolites and the status of asthma. In addition, multinomial logistic regression analysis was used to assess the exposure risk of DEHP among different phenotypic clusters and controls. Statistical analyses were performed using SPSS (IBM Corp. Released 2016. IBM SPSS Statistics for Windows, Version 24.0. Armonk, NY, USA) software, and *P*-values less than 0.05 were considered significant.

## Results

### Study participants

Table 1 illustrates the demographic differences between the case and control groups. Variables such as age, BMI, education, household income, atopy, asthma severity, FEV_1_%, FEV_1_ (L), PEFR%, and various asthma comorbidities showed significant differences. Notably, there were no significant differences in smoking status or exposure to second-hand smoke at home or work. This table was referenced from Wu et al., except for education and household income variables [23]. Cases had a mean age and BMI of 56.0 and 25.1 kg/m^2^, respectively, whereas controls had a mean age and BMI of 53.6 and 24.3 kg/m^2^, respectively. The results indicated that the education level and household income were higher in the control group compared to the case group. Among cases and controls, the percentage of atopic participants was 78.9% (*n* = 288/365) and 5.1% (*n* = 12/235), respectively. In the case group, 68.5% (250/365) had severe/very severe asthma, while 31.5% (115/365) had mild/moderate severity of the disease. The mean FEV_1_ and its %predicted and PEFR %predicted of cases and controls were 2.0 and 2.4 L, 78.2% and 87.6%, and 70.0% and 72.2%, respectively. The proportions of asthma with co-morbid conditions such as T2DM, hypertension, or a combination of T2DM and hypertension, as well as allergic rhinitis, were as follows: 12.3% (*n* = 45/365), 29.3% (*n* = 107/365), 7.4% (*n* = 27/365), and 66.0% (*n* = 241/365), respectively. Additionally, Table A.1 revealed the frequency of using plastic packaging for both food and drinks. Cluster E exhibited the highest frequency, followed by Clusters C, B, D, A, and F.

**Table 1:** Demographics of study participants (*n* = 600)

	Control	Case	*P*-value
N	235	365	
Gender, N (%)^a^			0.12
Male	111 (47.2)	149 (40.8)	
Female	124 (52.8)	216 (59.2)	
Age (yrs), Mean (± SD)^b^	53.6 (± 13.6)	56.0 (± 14.8)	0.01*
BMI (kg/m^2^), Mean (± SD)^b^	24.3 (± 3.4)	25.1 (± 4.1)	0.02*
Education, N (%)^a^			< 0.001***
College/University	75 (31.9)	60 (16.4)	
High school	160 (68.1)	305 (83.6)	
Household Income, N (%)^a^			0.001**
> 100 (thousand dollars)	9 (3.8)	10 (2.7)	
67 < x ≤ 99 (thousand dollars)	10 (4.3)	6 (1.6)	
34 < x ≤ 66 (thousand dollars)	44 (18.7)	44 (12.1)	
≤ 33 (thousand dollars)	80 (34.0)	102 (27.9)	
Unknown / Prefer not to disclose	92 (39.1)	203 (55.6)	
Onset age (yrs), Mean (± SD)^b^	NA	43.8 (± 20.0)	NA
Smoking status, N (%)^a^			0.15
Current smoker	181 (77.0)	283 (77.5)	
Ex-smoker	28 (11.9)	56 (15.3)	
Never smoker	26 (11.1)	26 (7.1)	
Second-hand Smoke at Home, N (%)^a^	158 (67.2)	227 (62.2)	0.21
Second-hand Smoke at Work, N (%)^a^	78 (33.2)	150 (41.1)	0.05
Atopy, N (%)^a^	12 (5.1)	288 (78.9)	< 0.001***
Asthma Severity, N (%)^a^			< 0.001***
Severe/Very severe	0 (0.0)	250 (68.5)	
Mild/Moderate	0 (0.0)	115 (31.5)	
ACT score, Mean (± SD)^b^	NA	20.6 (± 3.5)	NA
IgE (U/mL), Mean (± SD)^b^	NA	186.8 (± 245.3)	NA
Eosinophil Count (/cumm), Median (25-75th )^b^	NA	234.8 (± 224.5)	NA
FVC%, Mean (± SD)^b^	85.5 (± 14.8)	86.3 (± 18.5)	0.13
FVC (L), Mean (± SD)^b^	2.8 (± 0.9)	2.7 (± 0.9)	0.16
FEV_1_%, Mean (± SD)^b^	87.6 (± 15.0)	78.2 (± 21.5)	< 0.001***
FEV_1_ (L), Mean (± SD)^b^	2.4 (± 0.8)	2.0 (± 0.8)	< 0.001***
PEFR%, Mean (± SD)^b^	72.2 (± 22.3)	70.0 (± 31.3)	< 0.001***
PEFR (L/S), Mean (± SD)^b^	312.0 (± 132.7)	324.8 (± 131.9)	0.22
FEV_1_/FVC%, Mean (± SD)^b^	85.1 (± 9.7)	73.8 (± 11.9)	0.34
Asthma comorbid with T2DM			< 0.001***
Yes	0 (0.0)	45 (12.3)	
No	0 (0.0)	320 (87.7)	
Asthma comorbid with hypertension			< 0.001***
Yes	0 (0.0)	107 (29.3)	
No	0 (0.0)	258 (70.7)	
Asthma comorbid with T2DM and hypertension			< 0.001***
Yes	0 (0.0)	27 (7.4)	
No, only T2DM or hypertension	0 (0.0)	214 (58.6)	
No	0 (0.0)	124 (34.0)	
Asthma comorbid with allergic rhinitis			< 0.001***
Yes	0 (0.0)	241 (66.0)	
No	0 (0.0)	124 (34.0)	

NA, not applicable

^a^ Chi–square test

^b^ Mann–Whitney U test

* *P* < 0.05; ** *P* < 0.01; *** *P* < 0.001

### Description of DEHP metabolites

The concentrations of MEHP, MEHHP, and MEHP + MEHHP in urine samples, as well as the MEHHP/MEHP ratio, were presented in Table 2. The median concentration of MEHP (8.74 vs. 5.48 µg/g creatinine), MEHHP (20.63 vs. 11.86 µg/g creatinine), and MEHP + MEHHP (30.73 vs. 18.20 µg/g creatinine) in cases were significantly higher than in controls. However, there were no significant differences in the MEHHP/MEHP ratio between cases and controls (2.49 vs. 2.14). In comparison to controls, Clusters C and D exhibited significantly higher concentrations (median: 11.42 and 104.75, respectively, compared to 5.48). Additionally, Clusters A, C, D, and F demonstrated increased levels of MEHHP (median: 25.93, 20.70, 196.72, and 19.65, respectively, compared to 11.86) and MEHP + MEHHP (median: 39.16, 30.74, 301.47, and 25.01, respectively, compared to 18.20) when compared to the control group. However, no significant differences were observed in the MEHHP/MEHP ratio among these clusters or between the cases and controls.

**Table 2:** Description of urinary DEHP metabolites comparisons between cases and controls, and among 6 phenotypic clusters and controls

Urinary metabolites (µg/g creatinine)	Control		Case		Cluster A		Cluster B
	Median (IQR)		Median (IQR)		Median (IQR)		Median (IQR)
MEHP	5.48 (6.77)		8.74 (13.22)***		10.69 (10.40)		7.95 (8.92)
MEHHP	11.86 (11.34)		20.63 (21.38)***		25.93 (16.60)**		16.71 (13.44)
MEHP + MEHHP	18.20 (15.46)		30.73 (34.17)***		39.16 (35.26)**		26.35 (24.63)
MEHHP/MEHP	2.14 (2.20)		2.49 (2.55)		2.54 (3.82)		2.46 (2.02)
Urinary metabolites (µg/g creatinine)	Cluster C		Cluster D		Cluster E		Cluster F
	Median (IQR)		Median (IQR)		Median (IQR)		Median (IQR)
MEHP	11.42 (19.01)**		104.75 (482.75)*		10.71 (19.80)		5.84 (10.79)
MEHHP	20.70 (21.37)***		196.72 (893.00)***		20.34 (36.20)		19.65 (16.99)***
MEHP + MEHHP	30.74 (58.29)***		301.47 (1375.64)***		36.88 (40.15)		25.01 (26.89)**
MEHHP/MEHP	2.44 (2.01)		3.50 (2.78)		1.93 (7.89)		2.62 (2.26)
Urinary metabolites (µg/g creatinine)	*P*-value^a^		*P*-value^b^				
MEHP	< 0.001		< 0.001				
MEHHP	< 0.001		< 0.001				
MEHP + MEHHP	< 0.001		< 0.001				
MEHHP/MEHP	0.097		0.563				

^a^ Tested by Mann–Whitney U test

^b^ Tested by Kruskal–Wallis H test and Bonferroni correction

* *P* < 0.05; ** *P* < 0.01; *** *P* < 0.001

### Relationship between DEHP metabolites and asthma

Table 3 presents the risk of DEHP exposure in both case-control relationships and comparisons among phenotypic clusters and controls. After adjusting for age, BMI, atopy, education, and household income, the odds ratios (ORs) for MEHHP and MEHP + MEHHP in cases were significantly higher than those in controls (ORs = 4.49 and 3.50, respectively). The analysis from multinomial logistic regression revealed significantly higher ORs for MEHHP in various clusters compared to controls. Specifically, Clusters E (comprising young males and current smokers with early onset and low ACT scores), D (consisting of older non-smoking atopic females with high BMI and poor lung function), C (encompassing older ex-smoking males with second-hand smoke exposure), F (including young, atopic, non-smoker males with early onset), and A (comprising older non-atopic and non-smoker females with late-onset asthma) exhibited ORs of 3.42, 3.00, 2.94, 2.92, and 2.86, respectively. The ORs for MEHP + MEHHP were significantly elevated across all clusters compared to controls, with Clusters E, D, C, A, and F having ORs of 3.31, 2.95, 2.92, 2.83, and 2.75, respectively. Furthermore, the ORs for MEHP were notably higher in Clusters C and D compared to controls, with ORs of 1.78 and 1.70, respectively. Importantly, only Cluster B (mainly older atopic and non-smoker females) exhibited no significant association with DEHP exposure risks.

**Table 3:** Current asthma and its phenotypic clusters in relation to DEHP exposures

Urinary metabolites		Case vs. control (*n* = 80/33)			Cluster A/control (*n* = 14/143)			Cluster B/control (*n* = 23/143)			Cluster C/control (*n* = 13/143)	
		ORs	*P*-value^a^		OR	*P*-value^b^		OR	*P*-value^b^		OR	*P*-value^b^
Model 1	MEHP	1.39	0.07		1.57	0.01		1.26	0.11		1.78	< 0.001*
Model 2	MEHHP	4.49	< 0.001*		2.86	< 0.001*		1.90	0.003		2.94	< 0.001*
	MEHHP/MEHP	1.06	0.23		1.06	0.19		1.03	0.32		0.91	0.23
Model 3	MEHP + MEHHP	3.50	< 0.001*		2.83	< 0.001*		1.87	0.003		2.92	< 0.001*
Urinary metabolites		Cluster D/control (*n* = 5/143)			Cluster E/control (*n* = 4/143)			Cluster F/control (*n* = 21/143)				
		OR	*P*-value^b^		OR	*P*-value^b^		OR	*P*-value^b^			
Model 1	MEHP	1.70	0.001*		1.79	0.02		1.57	0.004			
Model 2	MEHHP	3.00	< 0.001*		3.42	< 0.001*		2.92	< 0.001*			
	MEHHP/MEHP	0.99	0.89		1.10	0.16		1.04	0.35			
Model 3	MEHP + MEHHP	2.95	< 0.001*		3.31	< 0.001*		2.75	< 0.001*			

^a^ Multiple logistic regression, adjusted for age, BMI, atopy, education, and household income; the significance level (α) was set at 0.05

^b^ Multinomial logistic regression, adjusted for education and household income; to reduce the potential for type I errors in our multiple comparison study, we set the α level to 0.00238095 (0.05/21) using the Bonferroni correction method

*The model exhibited statistical significance

Table 4 presents the relationship between DEHP exposure and different classifications compared to controls, based on asthma severity, atopy, asthma comorbidity with T2DM or hypertension or both, and asthma comorbidity with allergic rhinitis. After adjusting for age, atopy, education, and household income, the ORs for MEHHP and MEHP + MEHHP were significantly higher in the severe/very severe group compared to controls (ORs = 3.85 and 3.19) with similar results being observed in the mild/moderate group (ORs = 3.60 and 2.98). Additionally, after adjusting for age, BMI, education, and household income, the ORs for MEHP, MEHHP, and MEHP + MEHHP were significantly higher in the atopic asthma group compared to controls (ORs = 1.56, 2.81, and 2.69), with similar results observed in the non-atopic asthma group (ORs = 1.61, 3.19, and 3.04). Following adjustments for age, BMI, atopy, education, and household income, asthma patients—whether they had T2DM, hypertension, or both conditions simultaneously—demonstrated significantly lower exposure risks for both MEHHP and MEHP + MEHHP compared to the control group, as detailed in Table 4. Furthermore, after adjusting for age, BMI, atopy, education, and household income, the ORs for MEHHP and MEHP + MEHHP were significantly higher in the group with comorbidity of allergic rhinitis compared to controls (ORs = 19.98 and 10.47), with similar results being observed in the group without comorbidity of allergic rhinitis (ORs = 10.62 and 6.45).

**Table 4:** Asthma status classifications in relation to DEHP exposures

Urinary metabolites		Severe/very severe vs. control (*n* = 36/33)			Mild/moderate vs. control (*n* = 44/33)			Atopic asthma vs. control (*n* = 66/131)			Non-atopic asthma vs. control (*n* = 14/131)	
		ORs	*P*-value^a^		ORs	*P*-value^a^		ORs	*P*-value^b^		ORs	*P*-value^b^
Model1	MEHP	1.38	0.07		1.33	0.10		1.56	< 0.001*		1.61	0.002*
Model2	MEHHP	3.85	< 0.001*		3.60	< 0.001*		2.81	< 0.001*		3.19	< 0.001*
	MEHHP/MEHP	1.07	0.22		1.05	0.34		1.02	0.60		1.06	0.11
Model3	MEHP + MEHHP	3.19	< 0.001*		2.98	< 0.001*		2.69	< 0.001*		3.04	< 0.001*
Urinary metabolites		Asthma/T2DM vs. control (5/33)			Asthma without T2DMvs control (*n* = 75/33)			Asthma/HPTS vs. control (*n* = 14/29)			Asthma without HPTS vs. control (*n* = 66/29)	
		ORs	*P*-value^c^		ORs	*P*-value^c^		ORs	*P*-value^c^		ORs	*P*-value^c^
Model1	MEHP	1.18	0.51		1.41	0.06		1.11	0.63		1.43	0.07
Model2	MEHHP	4.40	< 0.001*		4.45	< 0.001*		4.50	0.001*		5.85	< 0.001*
	MEHHP/MEHP	1.10	0.16		1.06	0.27		1.14	0.04		1.06	0.33
Model3	MEHP + MEHHP	3.18	0.003*		3.51	< 0.001*		3.02	0.004*		3.95	< 0.001*
Urinary metabolites		Asthma/T2DM/HPTS vs. control (*n* = 1/29)			Asthma without T2DM & HPTS vs. control (*n* = 62/29)			Asthma/allergic rhinitis vs. control (*n* = 52/27)			Asthma without allergic rhinitis vs. control (*n* = 28/27)	
		ORs	*P*-value^c^		ORs	*P*-value^c^		ORs	*P*-value^c^		ORs	*P*-value^c^
Model1	MEHP	0.70	0.41		1.39	0.15		2.01	0.03		1.62	0.06
Model2	MEHHP	5.36	0.01*		7.02	< 0.001*		19.98	< 0.001*		10.62	< 0.001*
	MEHHP/MEHP	1.25	0.02		1.09	0.23		1.04	0.65		1.08	0.24
Model3	MEHP + MEHHP	2.85	0.07		4.20	< 0.001*		10.47	< 0.001*		6.45	< 0.001*

T2DM, Type 2 Diabetes Mellitus

HPTS, hypertension

^a^ Multinomial logistic regression, adjusted for age, atopy, education, and household income

^b^ Multinomial logistic regression, adjusted for age, BMI, education, and household income

^c^ Multinomial logistic regression, adjusted for age, BMI, atopy, education, and household income

*To minimize the risk of type I errors in our multiple comparison study, we set the α level to 0.01666667 (0.05/3) using the Bonferroni correction method

### Correlation between urinary DEHP metabolites to biomarkers

Table 5 (MEHP + MEHHP), Table 6 (MEHP), and Table 7 (MEHHP) show the results from the Spearman’s rank correlation analyses of urinary DEHP metabolites and oxidative stress, sphingolipid metabolites, and inflammatory biomarkers. This study combined the levels of MEHP and MEHHP to explore the differences in exposure to primary and secondary metabolites among different phenotypes and comorbid conditions. The concentration of the sum of primary and secondary metabolites (MEHP + MEHHP) showed a higher correlation with MEHHP than with MEHP, emphasizing the significance of MEHHP exposure among these subgroups.

**Table 5:** Correlation between urinary MEHP + MEHHP and a panel of inflammatory markers

Urinary DEHP metabolite	Stratification	Status	MEHP + MEHHP	MEHP	MEHHP	HEL	4-HNE	S1P	C1P	IL-1β	IL-6	IL-8	IL-10	IL-13	IL-17	IFN-γ	MCP-1	MIP-1β
			ρ	ρ	ρ	ρ	ρ	ρ	ρ	ρ	ρ	ρ	ρ	ρ	ρ	ρ	ρ	ρ
MEHP + MEHHP	Asthma	+	NA	0.84^***^	0.92^***^	0.19^*^				-0.24^**^	-0.31^***^	-0.22^**^		-0.20^**^	-0.22^**^	-0.21^**^	-0.18^*^	-0.22^**^
		-	NA	0.81^***^	0.94^***^	0.17^**^	0.27^***^											
	Asthma-phenotypic clusters	A	NA	0.75^***^	0.84^***^						-0.61^**^							-0.54^**^
		B	NA	0.78^***^	0.94^***^					-0.32^*^	-0.32^*^	-0.30^*^	-0.35^*^	-0.32^*^	-0.29^*^			
		C	NA	0.93^***^	0.95^***^													
		D	NA	0.78^***^	0.84^***^								0.39^*^					
		E	NA															
		F	NA	0.87^***^	0.97^***^													
	Asthma severity^a^	++	NA	0.88^***^	0.92^***^									-0.27^*^				
		+	NA	0.81^***^	0.92^***^			-0.26^*^		-0.40^***^	-0.50^***^	-0.35^***^			-0.37^***^	-0.38^***^	-0.30^**^	-0.38^***^
	Atopic	+	NA	0.85^***^	0.93^***^	0.21^*^				-0.21^*^	-0.27^**^	-0.19^*^		-0.19^*^	-0.22^*^	-0.17^*^		-0.17^*^
		-	NA	0.78^***^	0.87^***^						-0.54^**^					-0.38^*^		-0.48^**^
	Asthma/T2DM	+	NA	0.81^***^	0.98^***^						-0.66^**^							
		-	NA	0.85^***^	0.91^***^	0.26^**^				-0.22^**^	-0.27^***^	-0.20^*^		-0.22^**^	-0.21^*^	-0.21^*^	-0.18^*^	-0.20^*^
	Asthma/hypertension	+	NA	0.79^***^	0.86^***^										-0.29^*^			
		-	NA	0.86^***^	0.94^***^	0.24^*^		-0.26^*^		-0.26^**^	-0.37^***^	-0.31^***^		-0.30^***^	-0.19^*^	-0.21^*^		-0.30^***^
	Asthma/T2DM/hypertension	+	NA	0.85^***^	0.98^***^			-0.68^*^	-0.93^**^		-0.89^***^			0.69^*^				
		-	NA	0.85^***^	0.93^***^	0.27^**^		-0.25^*^		-0.25^**^	-0.35^***^	-0.30^**^		-0.28^**^	-0.19^*^	-0.21^*^		-0.31^**^
	Asthma/allergic rhinitis	+	NA	0.85^***^	0.93^***^					-0.20^*^	-0.26^**^	-0.21^*^		-0.19^*^	-0.21^*^	-0.21^*^		-0.22^*^
		-	NA	0.80^***^	0.89^***^			-0.53^**^		-0.33^*^	-0.44^**^							

NA, not applicable

^a^ ++, severe/very severe; +, mild/moderate

^*^P < 0.05; ^**^P < 0.01; ^***^P < 0.001

**Table 6:** Correlation between urinary MEHP and a panel of inflammatory markers

Urinary DEHP metabolite	Stratification	Status	MEHP + MEHHP	MEHP	MEHHP	HEL	4-HNE	S1P	C1P	IL-1β	IL-6	IL-8	IL-10	IL-13	IL-17	IFN-γ	MCP-1	MIP-1β
			ρ	ρ	ρ	ρ	ρ	ρ	ρ	ρ	ρ	ρ	ρ	ρ	ρ	ρ	ρ	ρ
MEHP	Asthma	+	0.84^***^	NA	0.63^***^	0.18^*^				-0.27^***^	-0.34^***^	-0.25^**^		-0.20^*^	-0.22^**^	-0.20^*^	-0.17^*^	-0.30^***^
		-	0.81^***^	NA	0.59^***^		0.22^***^											
	Asthma-phenotypic clusters	A	0.75^***^	NA			-0.54^**^				-0.69^***^			-0.44^*^	-0.48^*^			-0.48^*^
		B	0.78^***^	NA	0.58^***^					-0.36^*^		-0.35^*^	-0.29^*^					
		C	0.93^***^	NA	0.81^***^													
		D	0.78^***^	NA	0.43^*^								0.55^**^					
		E		NA														
		F	0.87^***^	NA	0.77^***^						-0.40^*^							
	Asthma severity^a^	++	0.88^***^	NA	0.69^***^	0.29^*^								-0.29^*^				
		+	0.81^***^	NA	0.59^***^					-0.41^***^	-0.48^***^	-0.39^***^			-0.30^**^	-0.31^**^	-0.24^*^	-0.45^***^
	Atopic	+	0.85^***^	NA	0.65^***^					-0.27^**^	-0.30^***^	-0.24^**^			-0.20^*^			-0.26^**^
		-	0.78^***^	NA	0.48^**^		-0.34^*^				-0.52^**^			-0.42^*^				-0.46^*^
	Asthma/T2DM	+	0.81^***^	NA	0.69^**^						-0.52^*^							
		-	0.85^***^	NA	0.63^***^	0.25^**^				-0.27^***^	-0.32^***^	-0.26^**^		-0.21^*^	-0.21^*^	-0.21^*^		-0.29^***^
	Asthma/hypertension	+	0.79^***^	NA	0.47^***^		-0.28^*^										-0.30^*^	
		-	0.86^***^	NA	0.68^***^					-0.31^***^	-0.37^***^	-0.35^***^		-0.29^**^	-0.18^*^			-0.38^***^
	Asthma/T2DM/hypertension	+	0.85^***^	NA	0.77^**^						-0.71^*^			0.67^*^				
		-	0.85^***^	NA	0.67^***^					-0.30^**^	-0.37^***^	-0.34^***^		-0.27^**^	-0.19^*^			-0.38^***^
	Asthma/allergic rhinitis	+	0.85^***^	NA	0.67^***^					-0.27^**^	-0.30^**^	-0.28^**^			-0.19^*^	-0.21^*^		-0.32^***^
		-	0.80^***^	NA	0.54^***^	0.33^*^					-0.45^**^			-0.36^*^	-0.30^*^			

NA, not applicable

^a^ ++, severe/very severe; +, mild/moderate

^*^P < 0.05; ^**^P < 0.01; ^***^P < 0.001

**Table 7:** Correlation between urinary MEHHP and a panel of inflammatory markers

Urinary DEHP metabolite	Stratification	Status	MEHP + MEHHP	MEHP	MEHHP	HEL	4-HNE	S1P	C1P	IL-1β	IL-6	IL-8	IL-10	IL-13	IL-17	IFN-γ	MCP-1	MIP-1β
			ρ	ρ	ρ	ρ	ρ	ρ	ρ	ρ	ρ	ρ	ρ	ρ	ρ	ρ	ρ	ρ
MEHHP	Asthma	+	0.92^***^	0.63^***^	NA					-0.17^*^	-0.20^*^	-0.16^*^		-0.17^*^	-0.16^*^			
		-	0.94^***^	0.59^***^	NA	0.16^*^	0.23^***^								0.14^*^			
	Asthma-phenotypic clusters	A	0.84^***^		NA													
		B	0.94^***^	0.58^***^	NA								-0.34^*^	-0.32^*^				
		C	0.95^***^	0.81^***^	NA													
		D	0.84^***^	0.43^*^	NA													
		E			NA													
		F	0.97^***^	0.77^***^	NA													
	Asthma severity^a^	++	0.92^***^	0.69^***^	NA													
		+	0.92^***^	0.59^***^	NA					-0.31^**^	-0.39^***^	-0.26^*^			-0.32^**^	-0.31^**^	-0.23^*^	-0.25^*^
	Atopic	+	0.93^***^	0.65^***^	NA	0.17^*^					-0.18^*^			-0.19^*^	-0.18^*^			
		-	0.87^***^	0.48^**^	NA			-0.56^*^				-0.37^*^						
	Asthma/T2DM	+	0.98^***^	0.69^**^	NA			-0.59^*^		-0.54^*^	-0.66^**^							
		-	0.91^***^	0.63^***^	NA								-0.19^*^	-0.19^*^				
	Asthma/hypertension	+	0.86^***^	0.47^***^	NA													
		-	0.94^***^	0.68^***^	NA	0.20^*^					-0.29^**^	-0.21^*^		-0.28^**^				-0.19^*^
	Asthma/T2DM/hypertension	+	0.98^***^	0.77^**^	NA			-0.68^*^			-0.90^***^			0.73^*^				
		-	0.93^***^	0.67^***^	NA	0.23^*^					-0.27^**^	-0.20^*^	-0.20^*^	-0.26^**^				-0.19^*^
	Asthma/allergic rhinitis	+	0.93^***^	0.67^***^	NA									-0.20^*^				
		-	0.89^***^	0.54^***^	NA			-0.55^**^		-0.31^*^								

NA, not applicable

^a^ ++, severe/very severe; +, mild/moderate

^*^P < 0.05; ^**^P < 0.01; ^***^P < 0.001

When stratified by asthma status, the correlation coefficients between MEHP + MEHHP and both MEHP (ρ = 0.84 vs. 0.81) and MEHHP (ρ = 0.92 vs. 0.94) were similar in both cases and controls. Clusters C and F consistently showed higher correlation coefficients between MEHP + MEHHP, MEHP, and MEHHP than Clusters A, B, and D. Specifically, for Cluster C, the correlations were MEHP + MEHHP vs. MEHP (ρ = 0.93), MEHP + MEHHP vs. MEHHP (ρ = 0.95), and MEHP vs. MEHHP (ρ = 0.81). For Cluster F, the correlations were MEHP + MEHHP vs. MEHP (ρ = 0.87), MEHP + MEHHP vs. MEHHP (ρ = 0.97), and MEHP vs. MEHHP (ρ = 0.77). In Cluster E, no significant correlations were observed among urinary DEHP metabolites. The correlation coefficients for MEHP + MEHHP vs. MEHP were ρ = 0.64, for MEHP + MEHHP vs. MEHHP were ρ = 0.64, and for MEHP vs. MEHHP were ρ = 0.26 (data not shown).

In the case group, we consistently observed significantly negative correlations between MEHP + MEHHP, MEHP, and MEHHP and IL-1β (ρ=-0.24, -0.27, and − 0.17), IL-6 (ρ=-0.31, -0.34, and − 0.20), IL-8 (ρ=-0.22, -0.25, and − 0.16), IL-13 (ρ=-0.20, -0.20, and − 0.17), and IL-17 A (ρ=-0.22, -0.22, and − 0.16). However, we found no significant correlation between these urinary DEHP metabolites and cytokines, except for the significantly positive correlation between MEHHP and IL-17 A (ρ = 0.14). Moreover, in Cluster B, we observed a significantly negative correlation between MEHP + MEHHP and MEHP to IL-1β (ρ=-0.32 and − 0.36), IL-8 (ρ=-0.30 and − 0.35), and IL-10 (ρ=-0.35 and − 0.29), respectively. Additionally, we found a significantly negative correlation between MEHP and IL-10 (ρ=-0.34).

We consistently observed significant positive correlations between the early lipid peroxidation product HEL and MEHP + MEHHP in both the case and control groups, as well as in the atopic group, asthma without T2DM group, asthma without hypertension group, and asthma without T2DM and hypertension group (ρ ranging from 0.17 to 0.27). Additionally, in the control group, we found a significant positive correlation between the late lipid peroxidation product 4-HNE and MEHP + MEHHP, MEHP, and MEHHP (ρ = 0.27, 0.22, and 0.23, respectively).

Table A.2 shows the Spearman’s rank correlation coefficients between urinary DEHP metabolites and total IgE levels. However, no significant positive or negative correlations were observed between urinary DEHP metabolites and total IgE levels.

## Discussion

### Summary

DEHP is a widely utilized plasticizer in plastic products. Research indicated that exposure to DEHP might have influenced asthma heterogeneity and associated comorbidities. To address this concern, our aim was to examine variations in DEHP exposure among different asthma phenotypes and comorbidities, utilizing primary and secondary DEHP metabolites. This investigation included the integration of a panel of physiological biomarkers to explore these associations. Emphasizing the impact of DEHP on asthma heterogeneity and related comorbidities, including potential alterations at the biological and molecular levels, suggested a new research direction distinct from previous studies.

### Exposure to DEHP stratified by asthma status

Numerous research findings indicate that the presence of urinary phthalate metabolite levels can serve as indicators of recent exposure to phthalates. However, these measurements may not accurately reflect long-term exposure due to the relatively brief half-lives associated with these chemical compounds [24, 25]. Nonetheless, given the practically continuous exposure, these chemicals are considered pseudo-persistent [24]. In the Taiwan study led by Tsai and colleagues, children’s urine revealed MEHP and MEHHP levels of 12.2 µg/g creatinine and 55.3 µg/g creatinine (baseline median) [26]. In contrast, a study by Zhang et al. in China found the geometric mean of MEHP in adult urine was 1.97 µg/g creatinine, and MEHHP was 6.44 µg/g creatinine [27]. In South Korea, Yoon et al.’s research showed the MEHHP level in the urine of the elderly was 25.93 µg/g creatinine [28]. Additionally, adults with current asthma in this study had median urinary levels of 8.74 for MEHP and 20.63 for MEHHP, while healthy controls had levels of 5.48 and 11.86 µg/g creatinine, respectively. These results highlighted higher plasticizer exposure in Taiwan compared to other countries.

The study results indicated that in various subgroups, the exposure risk of DEHP’s secondary metabolite, MEHHP, was higher than the sum of primary and secondary metabolites (MEHP + MEHHP). We found that the DEHP exposure risk in asthma patients (all asthma patients) was significantly higher than that in the phenotypic clusters (Clusters A to F). We also observed significant but varying levels of exposure differences among different asthma phenotypes. In these asthma phenotypes, the ORs of MEHHP consistently exceeded those of MEHP + MEHHP. MEHP showed the highest exposure risk in Cluster C, followed by D among different phenotypes. These results underscored DEHP exposure in Clusters E, C, and D.

Existing data suggested that differences in food packaging and individual variations may contribute to exposure variations among different asthma phenotypes. Moreover, alternative exposure routes, such as skin exposure (resulting from the use of medical and household products) and inhalation exposure (due to air pollution), could also contribute to variations in DEHP exposure among phenotypic clusters. Regarding food and drinks with plastic packaging, according to the results of the questionnaire survey, participants from Cluster E had the highest frequency of using plastic packaging for both food and drinks, followed by Clusters C, D, B, A, and F. Regarding individual variations, it is widely recognized that the ratio of MEHHP to MEHP (MEHHP/MEHP) serves as an indicator of the efficiency of DEHP’s secondary and primary metabolites. Although no significant differences were found between cases and controls or among different phenotypes and controls, we still observed that the median and mean of MEHHP/MEHP in asthma patients were slightly higher than those in the control group. The comparison between cases and controls showed marginal significance.

When stratified by asthma severity (severe/very severe vs. mild/moderate), we observed higher exposure risks of MEHHP and MEHP + MEHHP in the severe/very severe groups compared to the mild/moderate groups. However, after evaluating asthma control using the Asthma Control Test (ACT), we found no significant correlation between DEHP exposure and asthma control. When considering disease comorbidity, the ORs for asthma alone were higher than those for asthma comorbid with T2DM, hypertension, or the combination of T2DM and hypertension. However, in cases where asthma was comorbid with T2DM or hypertension, we observed that MEHP exposure was not associated with asthma. Additionally, we observed significantly higher exposure risks of MEHP, MEHHP, and MEHP + MEHHP in non-atopic asthma compared to atopic asthma. Notably, this was the only grouping where the exposure risk of MEHP reached statistical significance. In the grouping of asthma comorbid with rhinitis (yes vs. no), we observed very high ORs, particularly in asthma comorbid with rhinitis, where the ORs for MEHHP and MEHP + MEHHP exceeded 10, and the ORs for asthma alone were also significantly higher than in other groups.

The significant and varying levels of DEHP exposures in the results highlight an increased risk of MEHHP and MEHP + MEHHP exposure in specific asthma subgroups, underscoring the complex relationship with asthma, especially concerning health effects. Notably, rodent studies provided evidence that phthalates, functioning as adjuvants at environmentally relevant doses, can induce respiratory and inflammatory reactions when combined with an allergen [12]. Cellular investigations have revealed that phthalates have the potential to modify both innate and adaptive immune responses [12]. Additionally, adult asthma often accompanied with other health conditions. Asthma and T2DM were prevalent chronic diseases on the rise, potentially linked to mild systemic inflammation and the use of corticosteroids and other medications [13]. The current study results supported an association between DEHP exposure and asthma or immune responses, aligning with the findings of the referenced studies on the risk of DEHP exposure and asthma status. We acknowledge the limitation of measuring only one primary metabolite, MEHP, and one secondary metabolite, MEHHP. Importantly, we observed that the concentration of the combined primary and secondary metabolites (MEHP + MEHHP) exhibited a stronger correlation with MEHHP than with MEHP alone, emphasizing the significance of MEHHP exposure within these subgroups.

### DEHP exposures and a panel of physiological indicators in asthma patients

Interestingly, we observed a consistent and negative correlation between DEHP metabolites and the selected cytokines when examining multiple physiological indicators. However, it is crucial to note that this finding may be influenced by factors such as peripheral blood collection, other prominent environmental health factors, or the medication habits of current asthma patients, all of which could potentially impact their physiological regulation. This observation may also suggest exploring a new direction in understanding the relationship between DEHP exposure and physiological markers, potentially diverging from previous interpretations. To confirm these results, more studies of various types and larger sample sizes are required.

Despite using different classifications for asthma conditions, no significant correlation was found between total IgE levels and the levels of DEHP metabolites in urine. While the link between DEHP and total IgE was not significant, noteworthy associations emerged with asthma severity and specificity. As asthma is a heterogeneous disease, based on a comprehensive consideration of 18 demographic and clinical parameters, we categorized asthma patients into six different phenotypes. We observed that among asthma patients with different phenotypes, Clusters D (value of 25th, 50th, and 75th percentiles: 41.3, 138.5, and 298.0 U/mL) and F (36.8, 103.0, and 301.0 U/mL) exhibited relatively higher total IgE levels, indicating higher DEHP exposure risks, surpassed only by Cluster E of current smokers and Cluster C of ex-smokers. These results highlight the complex interplay between environmental exposure and asthma immunology.

It was also perplexing to note that most cytokines presented a significant negative correlation with DEHP concentration. Specifically, IL-1β, IL-6, IL-8, IL-13, and IL-17 in the case group showed consistent results. The reason for these findings is, at present, unclear. It could be that the circulating levels of cytokines may not be indicative of those at sites of tissue mucosa, or it might be that under certain circumstance, DEHP exposure may cause immune cell death, thereby reducing the levels of immune cell-derived cytokines. It is also likely that the primary DEHP metabolite, MEHP, known to activate PPARγ (peroxisome proliferator-activated receptor gamma), differentially regulate the immune response in the context- and cell-dependent manner. While these possibilities could not be discerned at this time, our recent research has revealed that environmental pollutants played a role in the development of allergic asthma and interact with innate immunity, holding significant importance [6]. Long-term exposure to DEHP promoted allergic lung inflammation, partly through the alteration of CD8α^+^ dendritic cells (DCs) differentiation via the MEHP-PPARγ axis. When mice were exposed to DEHP at a human tolerable daily intake dose, it led to changes in DCs in the spleen, DC progenitors in the bone marrow, decreased IL-12 production in splenic DCs, and increased T helper 2 polarization, it exacerbated allergic lung inflammation in mice. The upregulation of PPARγ enhanced the migration and Th2-priming capacity of lung DCs, indicating a pro-inflammatory role for PPARγ in Th2-mediated allergic lung inflammation [5]. Furthermore, systemic treatment with a pharmacological PPARγ agonist has shown to reduce inflammation, partly by inhibiting DC function in various inflammatory diseases, including asthma [29]. These findings supported the notion that PPARγ undergoes immune suppression following DEHP exposure, potentially altering the immune response in the body.

It was reasonable to explain that DEHP exposure in the participants of this study did not significantly worsen asthma symptoms (stratified by severity) or elicited an immediate response (correlation between DEHP exposure and a panel of inflammatory markers). It was also possible that their levels of DEHP exposure were not accurately reflected in total IgE levels. Our findings reveal a statistically significant negative correlation between urinary DEHP metabolites—whether MEHP, MEHHP, or MEHP + MEHHP—and various sphingolipids and cytokines. The unexpected negative correlation between DEHP and these biomarkers of inflammatory response contrasts with previous studies that hypothesized DEHP stimulates the inflammatory process [30]. However, a study using rat alveolar macrophages suggested that MEHP can induce both pro-inflammatory and anti-inflammatory responses [31]. Additionally, research on human lung epithelial cells indicated a non-linear, inverted U-shaped relationship between the effects of phthalates, including MEHP, on cytokines such as IL-6 and IL-8 [32]. Moreover, a mouse study found a significant negative correlation between ingesting DMP (Dimethyl phthalate, a plasticizer) and blood levels of IL-4, IL-6, and IFN-γ. The authors demonstrated that DMP confirms this finding through immune toxicity induced by oxidative damage and mechanisms such as cell apoptosis [33], aligning with recent epidemiological research results [34]. Although there is currently no conclusive explanation for the observed correlation between DEHP and cytokines in our study, and phthalates may share similar metabolic and toxicological mechanisms, the higher DEHP exposure in our study population compared to other studies cannot be ruled out. The potential impact of high exposure on immune toxicity requires further validation through subsequent relevant basic and epidemiological research.

Positive correlations were identified between urinary DEHP metabolite concentrations and HEL in various subgroups, with the control group displaying a significant positive correlation with 4-HNE. Despite the absence of a correlation between DEHP exposure and HEL in different asthma phenotypes, a connection was observed between DEHP exposure and elevated HEL levels in asthma patients without comorbidities such as T2DM, hypertension, or the combination of T2DM and hypertension. These findings indicate that HEL may serve as an early marker for lipid peroxidation and a more sensitive indicator of environmental exposures, including DEHP, in both asthma patients and healthy control groups. Elevated oxidative stress triggers enzymatic processes that enhance sphingolipid (SL) metabolism, generating bioactive lipid metabolites and crucial signaling mediators. Notably, sphingosine-1-phosphate (S1P) and ceramide-1-phosphate (C1P) play pivotal roles in regulating angiogenesis and inflammation. S1P, for instance, governs various inflammatory processes through its interaction with S1P receptors (S1PRs), influencing mast cell responses, airway smooth muscle contraction, and airway hyperreactivity. In the context of asthma comorbidities, the total of MEHP and MEHHP pose a co-exposure risk. The subsequent response, characterized by increased HEL and decreased S1P levels, serves as a common factor with varying degrees of risk, suggesting potential differences in regulatory mechanisms.

### Novelties and limitations

In summary, our discussion on the clinical significance of DEHP exposure in different asthma phenotypes and comorbidities involved a thorough consideration of various factors. We conducted a detailed analysis of asthma phenotypes, covering symptom severity, disease duration, and treatment response. Simultaneously, we assessed comorbidities like T2DM and hypertension to compare DEHP exposure risk with asthma alone, offering insights into its clinical significance for patients with multiple chronic conditions. We also investigated the impact of DEHP exposure on asthma control, using tools like the ACT to evaluate its effects on quality of life and treatment outcomes. When exploring genetic and immune differences among asthma phenotypes, we focused on individual characteristics influencing DEHP response, aiding in determining sensitivity and potential variations in different immune backgrounds. Considering the influence of dietary habits on DEHP exposure, particularly in foods and beverages with plastic packaging, our analysis of patients with different asthma phenotypes provided deeper insights into potential effects on DEHP concentrations. These comprehensive studies have provided a more holistic understanding of the clinical impact of DEHP exposure on various asthma phenotypes and comorbid patients, leading to more insightful research results and clinical recommendations. This study had several limitations. Firstly, being a case-control study, it was unable to establish direct causal relationships between DEHP exposures and physiological indicators. Secondly, the limited availability of information on asthma comorbidities among the study participants restricted the exploration of potential risk factors associated with DEHP exposure and its impact on asthma comorbidities. Additionally, the lack of detailed questionnaire data related to dietary habits was another limitation. Considering more potential confounders (education and household income), the sample size within certain clusters is quite small (Table 3 and 4), which may be mentioned among the study limitations.

## Conclusion

DEHP, a widely used plasticizer in plastic products, may influence asthma heterogeneity and associated comorbidities. To address this, we examined DEHP exposure variations across asthma phenotypes and comorbidities using primary and secondary DEHP metabolites. Integrating physiological biomarkers, we highlighted DEHP’s impact on asthma and comorbidities, signaling a unique research direction. Results revealed higher risk of DEHP’s secondary metabolite, MEHHP, in various subgroups, surpassing the sum of primary and secondary metabolites. Asthma patients, especially in Cluster E, using plastic packaging extensively, showed elevated DEHP risk. Asthma patients without comorbidities had higher DEHP ORs, and severe/very severe asthma exhibited higher DEHP ORs than mild/moderate cases. Notably, allergic rhinitis was linked to higher DEHP ORs. However, asthma patients without allergic rhinitis still had higher DEHP ORs, underscoring a complex relationship. We observed a consistent negative correlation between DEHP metabolites and selected cytokines across physiological indicators. It is essential to acknowledge potential influences, such as blood collection and environmental factors. This suggests an innovative research avenue, requiring further epidemiological and animal studies to explore the potential physiological effects of this phenomenon with more robust evidence.

### Electronic supplementary material

Below is the link to the electronic supplementary material.

Supplementary Material 1

## Data Availability

Not applicable.

## Abbreviations

DEHP: Di-(2-ethylhexyl) phthalate
HEL: Nε-(hexanoyl)-lysine
4-HNE: 4-hydroxynonenal
ACT: Asthma control test
MEHP: Mono(2-ethylhexyl) phthalate
MEHHP: Mono(2-ethyl-5-hydroxyhexyl) phthalate
LC-MS/MS: Liquid chromatography-tandem mass spectrometry
S1P: Sphingosine-1-phosphate
C1P: Ceramide-1-phosphate
