# Supplementary material for: Increased di-(2-ethylhexyl) phthalate exposure poses a differential risk for adult asthma clusters
Source: Respir Res. 2024 Mar 23;25:139. doi: 10.1186/s12931-024-02764-8 (PMC10960985; doi:10.1186/s12931-024-02764-8)
Supplement: Supplementary file 1 — Supplementary Material 1 [file 12931_2024_2764_MOESM1_ESM.docx]

**Increased di-(2-ethylhexyl) phthalate exposure poses a differential risk for adult asthma clusters**

| **Detail** | | |
| --- | --- | --- |
| Study design | Cross-sectional study | |
| Duration | Participants were recruited between 2011 and 2015 | |
| Location | Kaohsiung City, Taiwan | |
| Control group (N=235) | Undergoing routine checkups; without T2DM, hypertension, and other respiratory and allergic diseases | |
| Case group (N=365) | Current asthma attending clinical visits | |
| **Grouping** | | |
| 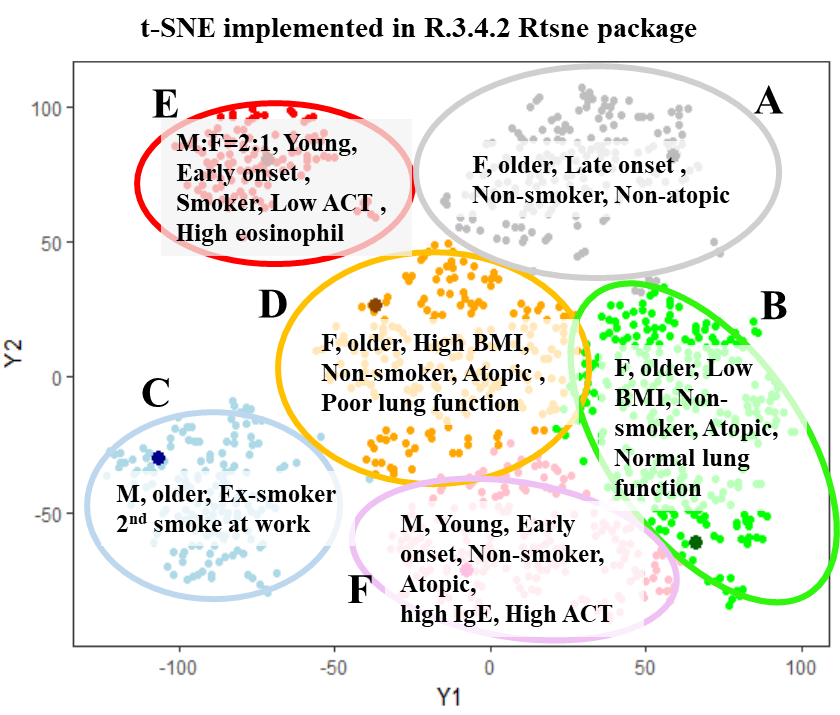A. Phenotypic clusters  Asthma phenotypic clustering was classified by t-SNE integrating 18 clinical, physiologic, pathologic, and demographic parameters. | | 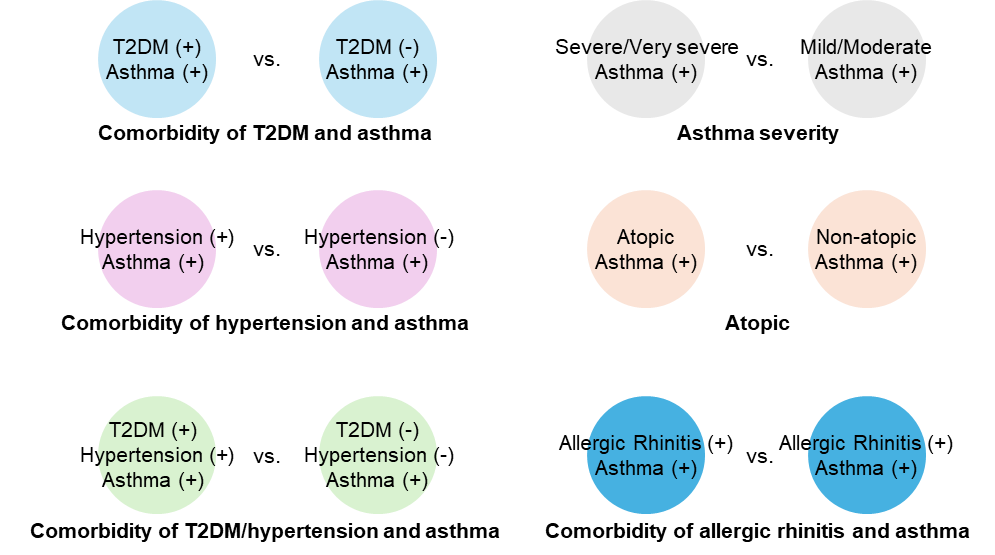B. Asthma with comorbidity |
| **Collection** | **Assessment** | |
| Urine | DEHP metabolites, such as MEHP and MEHHP; oxidative stress markers | |
| Plasma | Sphingolipid metabolites and inflammation markers | |
| Questionnaire | Demographic information, lifestyle factors, medical history, and medication records, among others | |


**Figure A.1**. Study Schematic.

**Table A.1.** Frequency of using plastic packaging for both food and drinks (N=600).

| Cluster | Plastic packaging for food  (times/week) | Plastic packaging for drinks  (times/week) |
| --- | --- | --- |
| A | 2 | 2 |
| B | 2 | 3 |
| C | 3 | 3 |
| D | 2 | 3 |
| E | 3 | 4 |
| F | 2 | 2 |

**Table A.2.** Correlation between total IgE and urinary DEHP metabolites.

|  |  | Urinary DEHP metabolites | | | | | | | |
| --- | --- | --- | --- | --- | --- | --- | --- | --- | --- |
|  |  | MEHP+MEHHP | |  | MEHP | |  | MEHHP | |
| Stratification | Status | ρ | P-value |  | ρ | P-value |  | ρ | P-value |
| Asthma | + | -0.01 | 0.94 |  | 0.02 | 0.79 |  | -0.05 | 0.50 |
|  | - | NA | NA |  | NA | NA |  | NA | NA |
| Phenotypic clusters of asthma | A | 0.02 | 0.91 |  | -0.09 | 0.68 |  | 0.10 | 0.64 |
|  | B | -0.07 | 0.62 |  | -0.08 | 0.60 |  | -0.13 | 0.36 |
|  | C | -0.01 | 0.96 |  | 0.10 | 0.58 |  | -0.09 | 0.60 |
|  | D | -0.04 | 0.82 |  | 0.19 | 0.31 |  | -0.17 | 0.38 |
|  | E | 0.21 | 0.61 |  | -0.05 | 0.91 |  | -0.31 | 0.46 |
|  | F | 0.08 | 0.67 |  | -0.01 | 0.95 |  | 0.09 | 0.61 |
| Severity^a^ | ++ | 0.02 | 0.86 |  | 0.09 | 0.45 |  | -0.04 | 0.71 |
|  | + | -0.03 | 0.77 |  | -0.03 | 0.75 |  | -0.04 | 0.69 |
| Atopic | + | 0.01 | 0.94 |  | 0.07 | 0.44 |  | -0.08 | 0.37 |
|  | - | 0.05 | 0.76 |  | -0.12 | 0.50 |  | 0.16 | 0.35 |
| Asthma/T2DM | + | -0.07 | 0.78 |  | -0.13 | 0.60 |  | -0.08 | 0.74 |
|  | - | 0.00 | 0.97 |  | 0.04 | 0.66 |  | -0.05 | 0.55 |
| Asthma/hypertension | + | -0.09 | 0.51 |  | -0.07 | 0.59 |  | -0.15 | 0.28 |
|  | - | 0.03 | 0.74 |  | 0.06 | 0.54 |  | 0.00 | 0.98 |
| Asthma/T2DM/hypertension | + | 0.13 | 0.70 |  | -0.14 | 0.66 |  | 0.15 | 0.65 |
|  | - | 0.06 | 0.53 |  | 0.08 | 0.39 |  | 0.03 | 0.79 |
| Asthma/allergic rhinitis | + | 0.01 | 0.88 |  | 0.05 | 0.58 |  | -0.06 | 0.51 |
|  | - | -0.07 | 0.63 |  | -0.06 | 0.64 |  | -0.01 | 0.93 |

NA, not applicable.

^a^ ++, severe/very severe; +, mild/moderate.
